# Poor construction, not time, takes its toll on subsidised fences designed to deter large carnivores

**DOI:** 10.1371/journal.pone.0175211

**Published:** 2017-04-10

**Authors:** Jens Frank, Ann Eklund

**Affiliations:** Grimsö Wildlife Research Station, Department of Ecology, Swedish University of Agricultural Sciences, Riddarhyttan, Sweden; University of Southern Queensland, AUSTRALIA

## Abstract

Large carnivore conservation may be considered as successful in Sweden, as wolf (*Canis lupus*), lynx (*Lynx lynx*), brown bear (*Ursus arctos*), golden eagles (*Aquila chrysaetos*), and wolverine (*Gulo gulo*) populations have recovered from extinction or near extinction to viable populations during the last three decades. Particularly the wolf and lynx populations have returned at the cost of an increasing number of carnivore attacks on domestic livestock. To support coexistence between carnivores and livestock production, the Swedish authorities subsidise interventions to prevent or reduce the number of carnivore attacks. The most commonly used intervention is carnivore deterring fencing, and all livestock owners can apply for subsidies to build a fence. To receive reimbursement the fence must be approved by the authorities according to predefined criteria. An important part of any management aiming to be adaptive is evaluating interventions. In this paper we evaluate to what extent previously subsidised fences still meet the criteria 1–15 years after their approval. Of 296 fences that had received subsidies in the county of Värmland, 100 randomly selected fences were revisited in 2016. From this subsample 14% of the fences still met the initial criteria for subsidies. None of the fences that still fulfilled the criteria were more than 8 years old, whereas fences with identified failures occurred in all age groups. Of the 86 fences that failed to meet the criteria, construction failures were the most commonly occurring problem. Maintenance failures, wear and tear, only explain a minor part of the failures. To improve the quality of fencing, as well as the quality and longevity of the subsidies programme, there is a need for improved communication between authorities, and improved communication and support from the authorities to livestock producers before and during construction of fences, as well as more rigorous inspection when the fences are built.

## Introduction

Throughout human history, wildlife species have suffered consequences of human dominance of the planet [1]. Recently a growing awareness and concern for biodiversity has led to more protection of threatened species globally. Conflict between human interests and wildlife is a widespread issue of growing concern for conservationists, not least in relation to large carnivores because they may kill both livestock and humans [2]. Conflicts between large carnivores and livestock owners can be mitigated through compensation payments for lost animals, i.e., post-conflict mitigation, or through subsidies for proactive measures such as fences, i.e., pre-conflict mitigation. The objective of compensation programs is to increase human tolerance towards wildlife [3]. However, compensation programs are often criticised as being inadequate, complicated, and expensive [4, 5, 6, 7]. In a Wisconsin study [8], it was concluded that, “compensation payments apparently do not improve individual tolerance toward wolves or people’s approval of lethal control”. In order to be more effective, compensation programs need to be a part of a comprehensive approach that includes options for control of offending animals, proactive mitigation measures, and, in some cases, broader financial incentives for changes in land use practices [9]. The latter approach has been used in India [10] and African countries [11]. Subsidies for proactive measures to reduce the risk of depredation from large carnivores are used worldwide. In Australia, state and federal funds are used to subsidise fencing to exclude dingoes (*Canis lupus dingo*) from vast sheep grazing areas [12]. In several European countries, e.g., Sweden, Norway, and Finland, as well as states in the U.S., e.g., Montana, Wyoming, Idaho, and Minnesota, subsidies are granted on a farm level to individual applicants, generally the livestock producers. Subsidies for farm-level proactive measures can include funding for purchasing and installing permanent electric fences, dogs to guard livestock, and help with temporary measures (e.g. fladry and sound scaring devices) directly after depredation to prevent repeated losses the following days [13]. Evaluations of the effectiveness of compensation programs are extremely rare and badly needed [9]. Evaluations of government programs that pay livestock producers for proactive measures are even fewer but equally necessary [10].

During the 20^th^ century, the native Swedish populations of lynx (*Lynx lynx*), wolves (*Canis lupus*), brown bears (*Ursus arctos*), golden eagles (*Aquila chrysaetos*), and wolverines (*Gulo gulo*) were pushed to extinction, or near extinction [14]. During the past three decades, their populations have been under legal protection and have recovered in their Swedish range [14]. Their recovery has come at a cost of conflict with human activities, particularly livestock farming, due to an increasing number of carnivore attacks on livestock, mainly sheep. Statistics from the Swedish Wildlife Damage Centre (SWDC) reveal that lynx and wolves cause the majority of livestock attacks, as their ranges overlap sheep farming areas to a greater extent than do brown bear, golden eagle, and wolverine [15]. Depredation events, where livestock are injured or killed by carnivores, can be traumatic for the afflicted ownerr. To ease the burden of livestock producers, the Swedish authorities issue economic compensation for animals lost to large carnivores [16]. Nevertheless, the compensation does not evade the risk of depredation, and proactive measures to prevent carnivore attacks are prioritised by the Swedish Environmental Protection Agency SEPA. Between 2006 and 2015, an average of €150.000 per year was spent on compensations, in comparison to an average of €1 million per year spent on proactive measures in Sweden [15].

The proactive measure to which most of the funds are allocated is carnivore deterring fencing [15]. It is possible for all livestock owners to apply for subsidies to cover 80% of the cost for building a large carnivore deterring fence. Funds are mainly allocated to farms in areas with high population densities of wolves and lynx. Sheep farms are prioritised over cattle and other livestock [17]. Reimbursement for the cost is made once the fence is built and approved by field personnel from the County Administrative Board (CAB). For approval, the fence must fulfil the criteria specified in predefined guidelines provided by the SWDC.

The national programme for subsidising large carnivore deterring fences started in 1997, and has become an important but expensive effort to facilitate coexistence between carnivores and human society. The guideline-criteria for large carnivore deterring fences are based on a) features believed necessary to deter large carnivores from entering pastures, and b) characteristics considered to increase the durability and ease of maintenance of the fence. Guideline criteria specify that electric fences should have at least 5 live wires with a voltage of at least 4500 volts at any time of the year, and the lowest wire must not be any higher than 30 cm above ground. The fences should also have robust corners and gates that allow the wire or net to have a high tension, all ditches that run under the fence should be filled or fenced, and the construction should have a large enough number of fence posts to keep lowest wire <30 cm above ground also when the terrain creates natural bends on the fence. To keep carnivores out, the fence must enclose all sides of the pasture. If these and a number of additional criteria are fulfilled, state subsidies can cover 80% of the cost for material and installation otherwise payed by the livestock owner. The fences are considered to have a life-expectancy of 10–15 years [18] if properly constructed and regularly maintained. So far, there has been no follow-up on to what extent the subsidised fences meet the initial criteria after years of use. For an adaptive management it is necessary to regularly evaluate the effect of interventions. In this study we aim to evaluate how well the subsidised fences met the initial criteria after up to 15 years of use. When fences did not meet the criterions we aimed to identify the most common issues. This is important knowledge when assessing the expected effectiveness and calculating the long-term running costs of this and other similar programmes.

## Methods

To be eligible for reimbursement, the fence must first have been inspected and authorised by field personnel from CAB. Therefore, the CAB keeps record of the fences that have been approved for subsidies from the programme. One of the longest records of carnivore fencing is found in the county of Värmland, where the main part of the Swedish wolf population, as well as a permanent lynx population, subsists. In 2016, the CAB of Värmland initiated an evaluation of previously subsidised fences. During the summer (May-August) of 2016, field personnel visited 100 farms that had received subsidies for large carnivore deterring fences between the years 2001 and 2015. Sampling was stratified in order to visit at least five farms with fences constructed each year (2001–2015). However, some years there were fewer than 5 five farms receiving subsidies. The CAB field personnel followed the same standardised protocol, and made the same on-site investigation, as when originally authorising fences for reimbursement. The investigation captured 30+ variables, ranging from critical issues such as the robustness of the construction, to details such as whether there are visible signs to warn the passers for electrical fencing. The field visits were part of the CAB authorities’ ordinary work with follow-up of subsidies. Applicants agree to the follow-up evaluation when signing the application and the investigators therefore needed no additional permissions to investigate the fences.

In this study we choose to focus on five variables that may be critical when it comes to fencing to deter large carnivores.

**Fig 1:**
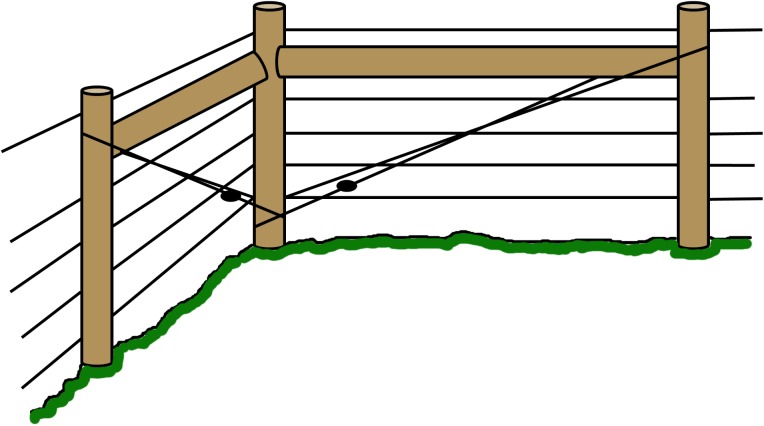
Robust corner. In order to meet the criterion for a robust corner, the corner must be locked tight by a wooden bar and hard-strung wire, made with poles of a minimum 15 cm diameter with their ends buried at a frost free depth, and with the top live wire running above the wooden bar.

**Fig 2:**
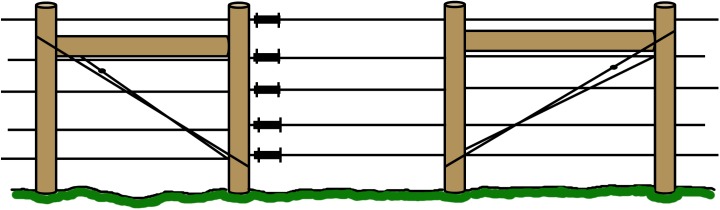
Robust ends. The fence “ends”, for instance by a gate, must be locked tight by a wooden bar and hard-strung wire, and built with robust posts of a minimum 15 cm diameter, in order to allow the live wires high tension.

**Fig 3:**
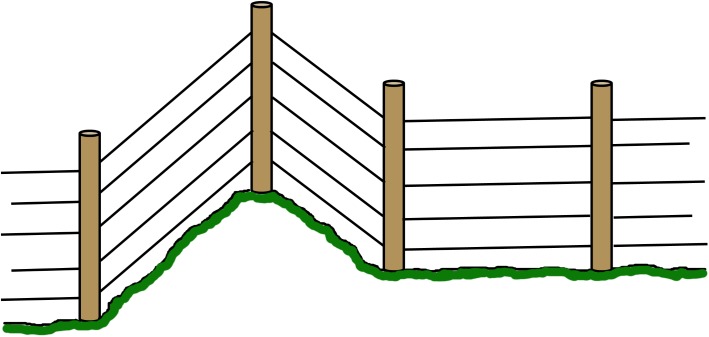
Variable terrain poles. Where the terrain is variable, such as when the fence runs across higher features and is forced to bend, poles must be placed in a suitable manner following the feature so that the lowest wire is never more than 30 cm above ground.

**Fig 4:**
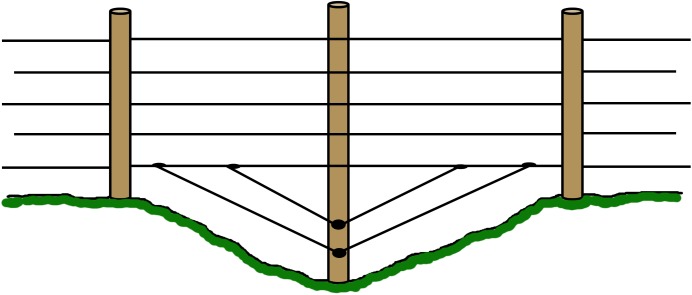
Ditches or gaps. Where the fence crosses a ditch or other features in the terrain which are acute enough to create a gap under the fence, a robust post should be placed at the lowest point onto which wires or a net should be attached to prevent carnivores from entering.

- Robust corners with a construction locked by a wooden bar and hard-strung wire, to allow high tension on the live wires (Fig 1). In the results we refer to failure of meeting this criterion as “corners”. Robust corners should last the life-time of the fence, and in instances where corners are missing it must thus be considered a construction failure.
- Robust ends with a construction locked by a wooden bar and hard-strung wire at gates and turn points to allow high tension on the live wires (Fig 2). In the results we refer to failure of meeting this criterion as “gates”. Robust ends should last the life-time of the fence, and in instances where robust ends are missing it is thus considered a construction failure.
- Posts placed at topographic bumps and bends to allow for the wire to stay parallel to the ground and prevent larger gaps between the lowest wire and the ground (Fig 3). In the results we refer to failure of meeting this criterion as “bends”. Posts in uneven terrain may have been sufficient when the fence was first approved, but annual maintenance is needed as the ground frost may push the posts up at the end of winter. The terrain may also change and create gaps under the fence. Therefore, failing bend posts is considered a maintenance failure.
- Lowest live wire <30 cm from ground to prevent large carnivores from crawling under the fence. In the results we refer to failure of meeting this criterion as “wire >30cm”. The height of the lowest wire may have been sufficient when the fence was first approved, but annual maintenance is needed as the ground frost may push up the posts and with them the wire, at the end of winter. It is of course also relatively easy to move the wire up or remove it entirely. Therefore, failing to keep the lowest wire <30 cm above ground is considered a maintenance failure.
- Ditches fenced or filled in order to keep a distance less than 30 cm from the lowest live wire to the ground to prevent large carnivores from walking or crawling under the fence (Fig 4). In the results we refer to failure of meeting this criterion as “ditches”. The fencing or filling of ditches may have been sufficient when the fence was first approved, but maintenance is needed to make sure that gaps are not created at these points. Therefore, failure to meet this criterion is considered a maintenance failure.

### Study area

The county of Värmland is located between 59°25′32″N and 13°16′17″E and covers an area of 17 583 km^2^. The main part of the study area is covered by boreal coniferous forests. The most common tree species are Norway spruce (*Picea abies*) and Scots pine (*Pinus sylvestris*), mixed with birch (*Betula pendula* and *B*. *pubescens*), aspen (*Populus tremula*) and alder (*Alnus incana* and *A*. *glutinosa*). From December to March, the ground is generally covered with snow of varying depth (5–50 cm). The area is characterized by intensive forestry with even aged forest stands, clear-cuts and a high density of forest roads. Pastures with grazing livestock constitute approximately 1% of the land surface. Sheep mainly graze in fenced pastures (less than 1% are free ranging). The number of sheep per farm is relatively small (92% of the sheep farms hold less than 50 ewes [19]. Large carnivores present in the area are lynx, brown bear, golden eagle, wolf and wolverine. The main wild prey species in the area are moose (*Alces alces*), roe deer (*Capreolus capreolus*), fallow deer (*Dama dama*), red deer (*Cervus elaphus*), badger (*Meles meles*), beaver (*Castor fiber*), brown hare (*Lepus europaeus*), mountain hare (*Lepus timidus*), capercaillie (*Tetrao urogallus*) and black grouse (*Tetrao tetrix*) [20].

## Results

**Fig 5:**
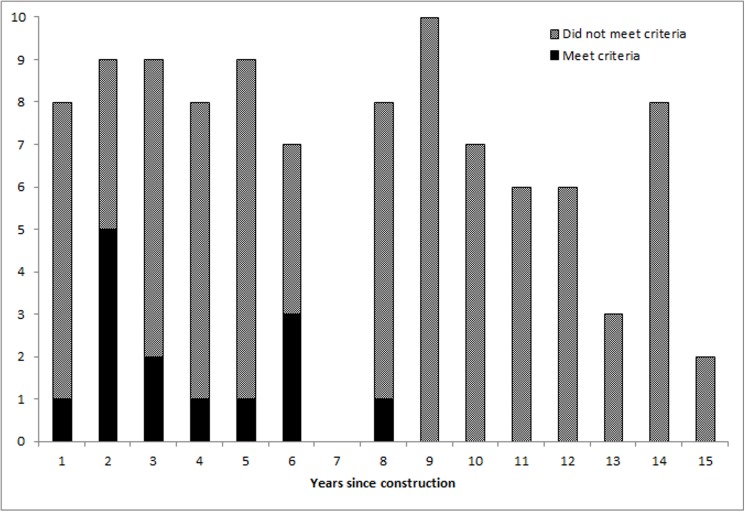
Fences meeting criteria versus age of fence. The number of fences that did, or did not, meet the criteria for subsidies in relation to the time since construction.

**Fig 6:**
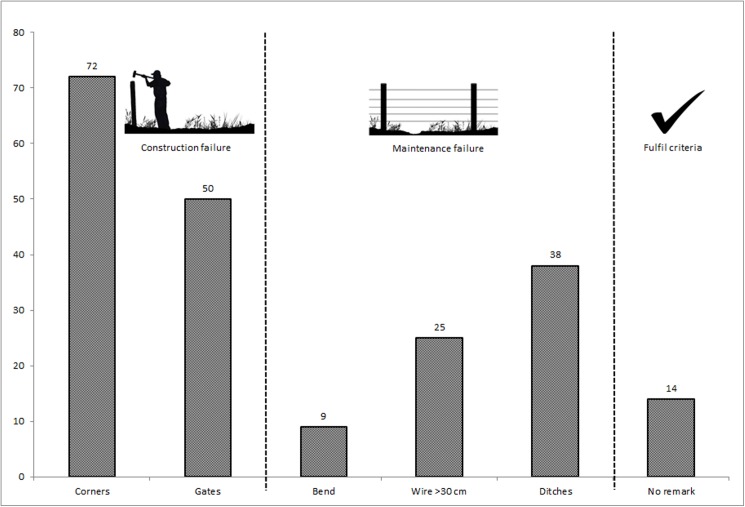
Construction or maintenance failures. Out of the surveyed fences (n = 100) that had originally been approved as carnivore deterring fences, n = 14 (i.e., 14%) still fulfilled the criteria, and n = 86 (i.e., 86%) failed to fulfil one or several criteria.

Between the years 2001 and 2015 the CAB of Värmland granted 296 applications for subsidised fences. From the randomly selected subsample (n = 100), 14% of the fences still met the initial criteria for subsidies. None of the fences that still fulfilled the criteria were more than 8 years old, whereas fences with identified failures occurred in all age groups (Fig 5). Out of the 86 fences that failed to meet the criteria, construction failures were the most commonly occurring problem (Fig 6).

## Discussion

Large amounts of tax money and other resources are spent on interventions intended to prevent human-wildlife conflicts. Since funds are limited it is important to follow up the resource use and identify ways to improve its effectiveness. In this study only a small proportion of subsidised fences met the criteria when revisited and inspected 1–15 years after their approval. No fence older than 8 years still met the criteria (Fig 5), indicating that wear and tear may be part of the explanation for why only 14% of the fences still meet the criteria. However, among the 43 fences approved and subsidised during the last five years, only 10 (23%) met the criteria. This suggests that many fences suffered from failures that should not originally have been approved.

Most failures that were identified for subsidised carnivore deterring fences in this study were actually related to construction failures rather than associated to wear and tear that would occur throughout the lifetime of the fence. Construction failures in fences older than 10 years (before 2006) may be explained by the absence of clearly written instructions for the CAB field personnel to follow during the assessments of fences. Since 2006, instructions have been present, but have either not been sufficiently communicated from the SWDC to the CAB field personnel or from the CAB field personnel to livestock producers applying for subsidies. The results highlight the importance of clear instructions and communication between the issuing authorities, i.e., SWDC and CAB´s, but also indicate a need for support to livestock producers that wish to construct new fences and apply for subsidies. If these producers are guided through the process, from the planning stage to finalisation of the fence construction, it is more likely that the fences will actually fulfil the criteria at inspection, and be sufficiently effective to protect the grazing livestock from carnivore attacks. With robust corners and ends by gates from the start on all locations, the durability of the entire programme would be greatly improved. Virtually all construction failures are avoidable in all locations. Maintenance failures are also possible to avoid but require continuous actions throughout the life span of the fence and are thus dependent on the owner´s own efforts. As owners are not compensated for the maintenance, it is difficult for authorities to exert control. Avoidance of construction failures on the other hand, should be considered as the responsibility of the authorities, as CAB field personnel visit all fences before subsidies are paid, to ensure that fences meet the specified criteria.

The county of Värmland has been the core area for the recovering Scandinavian wolf population since the first reproduction in the early 1980´s. The county thus has a longer history of managing depredation events compared to other counties in Sweden. All counties are however governed by the same legislation, funded from the same agencies, and obliged to follow the same protocols. Furthermore, the field personnel doing the investigations are trained in the same way in all counties and it is therefore likely that similar problems occur with regards to the fencing programme nationwide.

## Conclusions

Few fences met the stipulated criteria when revisited 1–15 years after construction. The most common reason for this was poor or improper construction, while lack of maintenance can only explain a small proportion of failures. Improved communications from the SWDC to CAB field personnel, increased communication and support from CAB to livestock producers before and during construction of fences, as well as more rigorous investigation when the fence is built, is needed and can be expected to drastically improve the quality of the subsidised fences. We suggest that the potential for improvement found in the Swedish system, with relatively small pastures that are easy to access by car, may also be found in other parts of the world where subsidised fences are larger and more difficult to reach.

## Supporting information

S1 File Complete dataset used in study. (XLSX)
